# Social Determinants of Health and Healthcare Access among Latina/x/o Sexual and Gender Minority Adults

**DOI:** 10.21203/rs.3.rs-5664699/v1

**Published:** 2024-12-30

**Authors:** Priya Mathur, Pedro Alonso Serrano, Gregory Phillips, Harita S. Shah

**Affiliations:** University of Chicago; Northwestern University; Northwestern University; University of Chicago

## Abstract

**Purpose:**

This study aims to identify current social determinants of health (SDOH) and healthcare access barriers impacting health outcomes among Latina, Latinx, and Latino (Latina/x/o) sexual and gender minority (SGM) individuals.

**Methods:**

We conducted cross-sectional surveys of 521 Latina/x/o adults with a focus on SGM individuals from November 2022 to June 2023 in Cook County, IL. We recruited using social media groups and in person community venues geared towards Latina/x/o and/or Lesbian/Gay/Bisexual/Transgender/Questioning (LGBTQ+) individuals. We examined associations between demographic predictors and SDOH (housing, food, and job insecurity) or healthcare access outcomes (insurance status, access to primary care, and time since last provider visit) using multivariable logistic regression.

**Results:**

Respondents included 68.8% (n=329) SGM individuals, of whom 48.6% (n=157) identified as gay or lesbian, 42.7% (n=138) identified as bisexual, pansexual, or queer, and 15.8% (n=48) identified as transgender. Across sexual and gender identities, our study population had a high burden of housing insecurity (n=286, 56.9%), food insecurity (n=177, 35.3%), and job insecurity (n=90, 27.8%). There were also a high proportion of uninsured individuals (n=120, 25.2%) and people who had not seen a provider in the last year (n=188, 36.2%).

**Conclusion:**

Public health interventions and policy reform are urgently needed to address the SDOH and healthcare barriers that drive health disparities for the diverse groups within Latina/x/o SGM populations.

## Background

Latina, Latinx, and Latino (hereafter referred to as Latina/x/o) populations have become the largest racial/ethnic minority in the U.S., making up 19% of the adult population, of whom an estimated 2,253,000 individuals identify as lesbian, gay, bisexual, or transgender [1, 2]. This growing population of Latina/x/o individuals who identify as sexual and gender minorities (SGM) experience intersecting forms of social marginalization (e.g., racism, heterosexism, cis-genderism), often leading to barriers to healthcare engagement and health disparities [3, 4]. However, few studies have focused on the drivers of health disparities among Latina/x/o SGM adults, a diverse population with unique and varied social and health-related needs [5, 6]. More research is needed to identify the SDOH driving health and healthcare disparities among Latina/x/o SGM populations, especially given recent shifts in immigrant populations and the growth of U.S.-born Latina/x/o populations [7, 8].

Social determinants of health (SDOH) such as housing insecurity, food insecurity, and job insecurity are known to have a significant impact on health outcomes and mortality [9–11]. For decades, SGM individuals have been shown to experience increased housing, food, and job insecurity related to adverse childhood experiences and social and structural discrimination [12–15]. Concomitantly, Latina/x/o individuals, especially those who are undocumented, experience high housing, food, and job insecurity relative to their non-Latina/x/o peers [16–23]. Many of these SDOH were exacerbated by the COVID-19 pandemic. Current assessments of SDOH for Latina/x/o SGM populations are urgently needed to guide public health research and policy [24–26].

In addition to socio-structural insecurities, Latina/x/o SGM populations also face barriers to healthcare engagement which exacerbate health disparities. Latina/x/o immigrants, particularly undocumented individuals, are more likely to experience lack of insurance, language barriers, and lack of a primary care home relative to their non-Latina/x/o peers [27–30]. Latina/x/o SGM individuals often experience discrimination and/or racism related to their intersecting identities that compound structural barriers to prevent engagement in healthcare [31, 32]. As a result, Latina/x/o SGM populations have historically lacked primary care access and been more likely to not have seen a provider in the last year relative to their non-Hispanic White peers [33]. While some research has shown that SGM individuals have relatively similar rates of being insured and accessing primary care relative to their cisgender and heterosexual counterparts, research is needed to explore healthcare access needs for Latina/x/o SGM individuals specifically [34].

In this study, we conducted a survey-based needs assessment to measure key SDOH and healthcare access among Latina/x/o SGM adults in Cook County, IL (including Chicago). Cook County, IL (including Chicago) is home to a large Latina/x/o SGM population, with 29% of the population identifying as Latina/x/o, approximately 19.2% of whom identify as lesbian, gay, bisexual, or transgender [7, 35]. We hypothesize that Latina/x/o SGM adults will have several unmet needs that may pose barriers to accessing and engaging in healthcare, and that the prevalence of these barriers may have increased post-pandemic. Understanding current SDOH and healthcare access needs for this population is critical to guiding interventions to reduce health and healthcare disparities.

## Methods

We conducted cross-sectional surveys between November 2022 and June 2023 to assess the needs of Latina/x/o adults with a focus on SGM individuals in Cook County, IL. Participants agreed to a written consent statement prior to participating. This study was determined exempt by the University of Chicago Institutional Review Board.

### Participants

Eligible participants self-identified as Hispanic or Latina/x/o, were 18 years of age or older, and lived in Cook County, IL. We tailored recruitment to reach a largely SGM population. We did not make SGM identity an inclusion criterion in order to understand SDOH prevalence in the context of the larger Latina/x/o population, and to avoid excluding individuals with same sex partners who may not identify as an SGM individual.

### Data Collection

Online recruitment entailed distribution of a Qualtrics link over Chicago social media groups centered on Latina/x/o or LGBTQ + members. Upon survey completion, respondents were provided with an option to refer their peers to participate. In-person recruitment occurred at Latina/x/o SGM-focused community events, community health centers, bars, and consulates. Surveys were available in English and Spanish and included multiple choice questions about demographic factors; sexual orientation; gender identity; health literacy; English proficiency; food, job, and housing insecurity; and healthcare access measures of insurance coverage, access to primary care, and time since last provider visit. Survey respondents were offered a $15 gift card for their participation.

### Measures

#### Food Insecurity

Participants reported food insecurity based on the question, “In the last 12 months, did you and/or other adults in your household ever cut the size of your meals or skip meals because there wasn’t enough money for food?” Responses of “Yes” were categorized as “Food Insecure,” while responses of “No” were categorized as “Food Secure” [36].

#### Job Insecurity

Participants reported job insecurity based on the question, “Thinking about the next 12 months, how likely do you think it is that you will lose your job or be laid off?” with responses of “Very likely,” “Fairly likely,” “Not too likely,” “Not at all likely,” “I will be leaving the labor force voluntarily,” “I am not currently employed,” and “Don’t know.” Responses of “Very likely” and “Fairly likely” were categorized as “Job Insecure,” and responses of “Not too likely” and “Not at all likely” were categorized as “Job Secure” [37]. Responses of “I will be leaving the labor force voluntarily,” “I am not currently employed,” and “Don’t know” were dropped from this analysis.

#### Housing Insecurity

Participants reported housing insecurity based on the question, “How often in the past 12 months would you say that you were worried or stressed about having enough money to pay your rent/mortgage?” with options of “Always,” “Usually,” “Sometimes,” “Rarely,” and “Never.” Responses of “Always,” “Usually,” and “Sometimes” were categorized as “Housing Insecure,” with all other responses categorized as “Housing Secure” [38].

#### Insurance Coverage

Insurance status was assessed using the question, “What is your insurance status?” with options of “I do not currently have health insurance,” “I have Medicaid,” “I have Medicare,” and “I have private health insurance.” Responses of “I do not currently have health insurance” were categorized as “Uninsured,” and any of the other responses to this question were categorized as “Insured” [39].

#### Access to Primary Care

Access to primary care was assessed using the question, “What kind of place do you go most often if you are sick and need health care?” with responses of “A doctor’s office or health center,” “Urgent care center or walk-in clinic in a pharmacy or grocery store,” “Emergency room,” “VA Medical Center or VA outpatient clinic,” “I do not go to one place most often,” and “Other.” Responses of “A doctor’s office or health center,” “Urgent care center or walk-in clinic in a pharmacy or grocery store,” or “VA Medical Center or VA outpatient clinic” were categorized as “Primary Care Access,” while any of the other responses to this question were categorized as “No Primary Care Access” [40].

#### Time Since Last Provider Visit

Participants reported the time since their last provider visit via the question, “How long has it been since you last saw a doctor or other health professional for a primary care visit, physical, or regular check-up?” with options of “Never,” “Within the past year,” “1–2 years ago,” “2–3 years ago,” “3–5 years ago,” “5–10 years ago,” and “Over 10 years ago.” Responses of “Within the past year” were categorized separately from all other responses [40].

#### Analysis

We compared socio-demographics, SDOH, and healthcare access outcomes between respondents who identified as SGM versus those who identified as heterosexual and cis-gender. We examined associations between demographic factors and SDOH or healthcare access outcomes using multivariable logistic regression. Given the tremendous within SGM populations, we conducted sub-analyses by SGM subgroups (e.g., bisexual individuals, transgender women) to assess for differences in SDOH or healthcare access outcomes. All multivariable logistic regression models included covariates of age and education status. We tested associations with race and ultimately did not include it in the models given incomplete responses; many participants did not respond or chose “other” rather than the defined racial categories, a pattern consistent with national trends in race reporting among Latina/x/o populations [41]. All analyses were conducted in STATA version 18 [42].

## Results

Surveys were completed by 521 participants between November 1, 2022, and June 30, 2023. Participant demographics are presented in Table 1 by sexual orientation and gender identity. Most survey respondents identified as SGM (n = 329, 68.8%), and many were aged 25–34 years (n = 253, 48.8%). About half of SGM respondents identified as gay or lesbian (n = 157, 48.6%) and 42.7% (n = 138) identified as bisexual, pansexual, or queer. SGM respondents were more likely to be from the U.S., be more educated, and have higher English proficiency and health literacy relative to the non-SGM respondents.

Across sexual orientation and gender identities, a high proportion of participants experienced housing insecurity (n = 286, 56.9%), food insecurity (n = 177, 35.3%), and job insecurity (n = 90, 27.8%) (Table 2). In respect to identifying priority subgroups, people ages 25–34 years were significantly more likely to be housing insecure, food insecure, and job insecure relative to people ages 18–24 years. Even with a small number of respondents (n = 25), Black or African American individuals were significantly more likely to be food and job insecure relative to their White counterparts. Non-binary individuals experienced significantly higher odds of job insecurity relative to cisgender men. Transgender men appeared to experience higher odds of housing, food and job insecurity relative to their cisgender male counterparts, though the small sample size (n = 11) precluded significance. Gay, lesbian, bisexual, pansexual, and queer individuals also demonstrate a trend towards significance in being more likely to experience housing and job insecurity, but less likely to experience food insecurity than their heterosexual counterparts.

In terms of healthcare access measures, a high proportion of participants did not have insurance (n = 120, 25.2%) and had not seen a provider in the last year (n = 188, 36.2%) (Table 3). There were no significant associations between sexual or gender identity and healthcare access outcomes. Uninsured participants were more likely to be from Mexico or Venezuela, be between ages 35–44 years, have limited health literacy, and have limited English proficiency. Importantly, most of the survey respondents did have access to primary care, even for those without insurance. A key exception was that immigrants from Venezuela were 11 times as likely to lack primary care access compared to people from the U.S. Limited English proficiency was also a significant predictor for lack of primary care access. People with a bachelor’s degree or higher education were significantly less likely to experience healthcare access issues (lack of insurance, no primary care access, lack of a visit with a provider in the last year) relative to people with a high school education or less.

## Discussion

Our findings demonstrate high rates of housing, food, and job insecurity among this urban Latina/x/o population with a focus on sexual and gender minority adults. Housing insecurity was markedly high across the study population at 56.9%, highlighting a need for urgent housing infrastructure and policy reform. Our study sample also experienced barriers to accessing and engaging in healthcare, as shown by the proportion of uninsured individuals and people who had not seen a provider in the last year. Latina/x/o SGM populations are often prioritized for health disparities initiatives including in the fields of HIV, mental health, and cardiovascular disease [43–45]. Our findings highlight the need for public health interventions and policy reform to address these SDOH and healthcare access barriers in order to effectively advance the health of Latina/x/o SGM populations.

The Latina/x/o SGM adults in our sample experienced high levels of housing, food, and job insecurity, consistent with previously studied Latina/x/o SGM populations and at higher rates than those of non-Latina/x/o White SGM populations [46]. Within Latina/x/o SGM populations, we identified specific subgroups that may be at particular risk for housing, food, and job insecurity. Young adults aged 25–34 years was significantly more likely to experience housing, food, and job insecurity relative to other age groups. This age group is also known to experience the highest HIV incidence and elevated suicide rates among Latina/x/o populations [47, 48]. As housing, food and job insecurities have been shown to result in psychological strain, substance use, and sexual risk taking, HIV and mental health interventions for Latina/x/o populations must address these SDOH in order to effectively improve outcomes [49–51]. Nonbinary individuals and transgender men also appeared to have at elevated odds of housing, food and job insecurity. While the granularity of our sexual and gender identity data may have limited significance in subgroup analysis, our research highlights the need for dedicated SDOH-focused research in these often understudied groups.

Regardless of sexual orientation or gender identity, the high rates of housing, food, and job insecurity in our sample corroborate prior research which found increases in these insecurities among Latina/x/o populations post-pandemic. A recent study of Latina/x/o individuals in Cincinnati found that 60% experienced food insecurity and 35% experienced housing insecurity post-pandemic, at rates significantly increased from pre-pandemic times [5]. Our findings similarly found high prevalence of housing, food, and job insecurity with a greater emphasis on housing insecurity (57% experiencing housing insecurity and 35% experiencing food insecurity), potentially driven by increasing housing costs and local housing policies. In our sample, Spanish-speaking immigrants were more likely to experience high housing, food, and job insecurity consistent with prior research [52, 53]. Additionally, Afro-Latinos in our sample were three times more likely to experience food insecurity and almost six times more likely to experience job insecurity compared to their White counterparts. Studies have found that non-Hispanic Blacks are more likely to experience food and job insecurity than non-Hispanic Whites, but few have focused on Afro-Latinos who often face marginalization or discrimination related to experiences from intersectional identities [54, 55]. Moreover, many Afro-Latinos, Caribeños, and mixed race individuals often do not identify as Black, which may lead to underestimates of these SDOH [56]. Research and policy reform is urgently needed to address the impacts of housing, food and job insecurity on the health of these often multiply marginalized Latina/x/o populations.

In terms of healthcare access, lack of insurance and lack of a provider visit in the past year were common issues in our study population. While the SGM individuals we recruited were more highly educated and English proficient, many still lacked insurance (21.2%, n = 65) and had not seen a provider in the past year (32.2%, n = 106). These findings reflect known barriers to care for SGM individuals, such as anticipated or day-to-day discrimination which can be a deterrent to healthcare engagement [31, 57]. There were no significant differences in insurance status, primary care access, or last provider visit by SGM identity, in contrast to prior research showing that SGM individuals experience greater barriers to healthcare access [58]. This discrepancy likely reflects the barriers encountered by the non-SGM Latina/x/o participants in our study, namely Spanish-speaking immigrants who often experience language or documentation barriers to care [58]. Respondents with limited English proficiency were significantly more likely to lack insurance and primary care access relative to individuals with English proficiency. Our findings highlight the importance of addressing uninsurance rates and barriers to healthcare engagement for Latina/x/o SGM adults, particularly immigrants, who face compounded challenges related to their intersectional identities.

### Limitations

The contributions of this study are not without limitations. First, we leveraged partnerships with Latina/x/o-focused community-based organizations to recruit a diverse sample of SGM participants. Because of this, we may have had limited reach to SGM Latina/x/o individuals who are not connected to community-based organizations. We worked to mitigate potential selection bias by also conducting dedicated outreach at community events, bars, and consulates outside of community-based organization partnerships. Second, the cross-sectional nature of this survey limits the conclusions we can draw in terms of causality and trends over time. The needs assessment nature of this project is meant to highlight priority areas for robust longitudinal research and future tailored interventions. Third, our data did have missing responses for the variables of race and job insecurity, which may have limited significance of analyses. We included respondents with these incomplete responses to ensure their experiences with the other SDOH and healthcare access outcomes were still incorporated, and more research is needed to better assess these measures in Latina/x/o populations. Lastly, the small size of subgroups of SGM individuals may have precluded significant conclusions, yet it allowed us to identify potential priority populations for health disparities research while avoiding the Latina/x/o monolith. Future research should include dedicated recruitment of Latina/x/o transgender and other SGM subgroups to identify how to address SDOH-related health disparities at a more tailored level to reflect their unique experiences.

## Conclusion

Latina/x/o SGM populations encounter SDOH and barriers to healthcare access that are critical factors in their health. Our study of Latina/x/o SGM populations found high housing, food, and job insecurity, as well as barriers to healthcare access. Given the pervasive impact of these factors, policy change and healthcare reform will be essential to efforts to achieve health equity. Our findings also illustrate the tremendous diversity in identities within Latina/x/o populations and the SDOH they experience. Tailored community-engaged research remains key to advancing the health of specific Latina/x/o communities. Our data can inform urgently needed public health interventions to address the SDOH and multifaceted barriers to healthcare that drive health disparities for Latina/x/o populations.

## Figures and Tables

**Table 1:** Demographic Characteristics by Sexual Orientation and Gender Identity

	Sexual/Gender Minority (N, column %) N = 329	Heterosexual and Cisgender (N, column %) N = 149	Chi-Squared Test p-value
**Age Categories (Years)**			0.024
18–24	79 (24.0)	34 (22.8)	
25–34	179 (54.4)	65 (43.6)	
35–44	41 (12.5)	24 (16.1)	
45+	30 (9.1)	26 (17.4)	
**Race**			<0.001
White	96 (29.2)	41 (27.5)	
Black or African American	16 (4.9)	7 (4.7)	
Native American or Alaskan Native	36 (10.9)	4 (2.7)	
Asian	4 (1.2)	0 (0)	
Native Hawaiian or other Pacific Islander	1 (0.3)	0 (0)	
Mixed	25 (7.6)	3 (2.0)	
Other	85 (25.8)	69 (46.3)	
Did not respond	66 (20.1)	25 (16.8)	
**Origin**			<0.001
United States	211 (65.1)	57 (40.1)	
Puerto Rico	4 (1.2)	0 (0)	
Mexico	80 (24.7)	39 (27.5)	
Venezuela	7 (2.2)	34 (23.9)	
Other	22 (6.8)	12 (8.5)	
**Education**			<0.001
Less than high school	24 (7.3)	16 (10.7)	
Completed high school	159 (48.5)	104 (69.8)	
Bachelor’s degree	91 (27.7)	23 (15.4)	
Advanced degree	54 (16.5)	6 (4.0)	
**Gender Identity**			<0.001
Cisgender Men	156 (54.9)	79 (53.0)	
Cisgender Women	64 (22.5)	70 (47.0)	
Nonbinary	36 (12.7)	0 (0)	
Transgender Men	11 (3.9)	0 (0)	
Transgender Women	17 (6.0)	0 (0)	
**Sexual Orientation**			<0.001
Asexual	12 (3.7)	0 (0)	
Bisexual, Pansexual, Queer	138 (42.7)	0 (0)	
Gay, Lesbian	157 (48.6)	0 (0)	
Straight (Heterosexual)^a^	8 (2.5)	149 (100)	
Not sure	8 (2.5)	0 (0)	
**English Proficiency**			<0.001
English Proficient	253 (91.0)	94 (66.7)	
Limited English Proficiency	25 (9.0)	47 (33.9)	
**Health Literacy Level**			<0.001
High	280 (85.1)	93 (62.8)	
Low	49 (14.9)	55 (37.2)	
**Housing Insecurity**			0.094
Housing Secure	126 (39.4)	70 (47.6)	
Housing Insecure	194 (60.6)	77 (52.4)	
**Food Insecurity**			0.052
Food Secure	216 (67.5)	85 (58.2)	
Food Insecure	104 (32.5)	61 (41.8)	
**Job Insecurity** ^b^			0.22
Job Secure	159 (70.7)	66 (77.6)	
Job Insecure	66 (29.3)	19 (22.4)	
**Insurance Status**			0.089
Insured	241 (78.8)	97 (71.3)	
Uninsured	65 (21.2)	39 (28.7)	
**Primary Care Access**			0.061
Have access	256 (96.2)	97 (91.5)	
Do not have access	10 (3.8)	9 (8.5)	
**Last Provider Visit**			0.20
Within the past year	223 (67.8)	92 (61.7)	
> 1 year ago or Never	106 (32.2)	57 (38.3)	

a. 8 of the heterosexual identifying individuals reported being transgender.

b. The total n for job insecurity was 324, as 197 respondents did not answer the job insecurity question. These respondents were included in the overall analysis as reports of other insecurities or healthcare access issues remain important.

**Table 2:** Housing, Food, and Job Insecurities by Demographics

Demographic Group	Housing Insecure (N, row %)^a^	aOR (95% CI)^b,c^	Food Insecure (N, row %)	aOR (95% CI)	Job Insecure (N, row %)^d^	aOR (95% CI)
Total N = 521	N = 286 (56.9)		N = 177 (35.3)		N = 90 (27.8)	
**Age Categories (Years)**						
18–24	58 (48.3)	1 (reference)	39 (32.5)	1 (reference)	11 (14.9)	1 (reference)
25–34	155 (63.0)	2.07** (1.31,3.28)	95 (38.5)	1.67* (1.03, 2.69)	49 (29.3)	3.05** (1.45, 6.43)
35–44	39 (54.9)	1.43 (0.79, 2.61)	23 (32.4)	1.12 (0.59, 2.13)	20 (41.7)	4.76** (1.98, 11.5)
45+	33 (50.8)	1.11 (0.59, 2.10)	19 (30.7)	0.85 (0.43, 1.70)	10 (28.6)	2.04 (0.75, 5.55)
**Race**						
White	78 (53.4)	1 (reference)	44 (30.6)	1 (reference)	16 (16.3)	1 (reference)
Black or African American	13 (54.2)	0.82 (0.33, 2.00)	15 (62.5)	2.87* (1.14, 7.19)	8 (53.3)	5.97** (1.80, 19.8)
Native American/Alaskan Native	26 (66.7)	1.68 (0.79, 3.60)	14 (35.9)	1.24 (0.58, 2.68)	7 (23.3)	1.62 (0.57, 4.61)
Asian	3 (75.0)	2.26 (0.22, 22.8)	1 (25.0)	0.76 (0.07, 8.01)	0 (0)	n/a
Native Hawaiian/Pacific Islander	1 (50.0)	0.89 (0.05, 15.1)	0 (0)	n/a	0 (0)	n/a
Mixed	17 (63.0)	1.51 (0.63, 3.60)	6 (22.2)	0.74 (0.27, 2.03)	6(31.6)	2.43 (0.77, 7.70)
Other	98 (60.9)	1.28 (0.80, 2.05)	60 (37.3)	1.22 (0.74, 2.00)	30 (30.9)	2.11* (1.03, 4.34)
Did not respond	50 (50.0)	0.74 (0.43, 1.26)	37 (37.0)	1.07 (0.61, 1.88)	23 (38.3)	2.80* (1.25, 6.28)
**Origin**						
United States	154 (56.4)	1 (reference)	85 (31.0)	1 (reference)	40 (20.3)	1 (reference)
Puerto Rico	3 (75.0)	2.09 (0.21,21.0)	2 (50.0)	1.86 (0.25, 13.9)	2 (50.0)	3.42 (0.43, 27.0)
Mexico	66 (51.6)	0.83 (0.52, 1.32)	31 (24.8)	0.77 (0.45, 1.30)	27 (32.9)	1.85 (0.99, 3.44)
Venezuela	35 (72.9)	1.69 (0.81,3.53)	36 (75.0)	6.07** (2.80, 13.2)	9 (69.2)	6.61** (1.77, 24.7)
Other	22 (62.9)	1.33 (0.63, 2.82)	18 (51.4)	3.02** (1.40, 6.50)	10 (50.0)	3.97** (1.45, 10.8)
**Education**						
Less than high school	31 (55.4)	1 (reference)	23 (41.8)	1 (reference)	6 (37.5)	1 (reference)
Completed high school	167 (60.1)	1.17 (0.63, 2.16)	113 (40.9)	0.90 (0.48, 1.67)	55 (32.9)	0.81 (0.26, 2.49)
Bachelor’s degree	58 (53.2)	0.77 (0.38, 1.54)	32 (29.1)	0.47* (0.23, 0.97)	21 (22.6)	0.40 (0.12, 1.35)
Advanced degree	30 (50.9)	0.65 (0.30, 1.41)	9 (15.3)	0.20** (0.08, 0.50)	8 (17.0)	0.27 (0.07, 1.05)
**Gender Identity**						
Cisgender Men	136 (56.2)	1 (reference)	85 (35.0)	1 (reference)	44 (26.2)	1 (reference)
Cisgender Women	72 (49.3)	0.86 (0.56, 1.32)	38 (26.2)	0.67 (0.42, 1.08)	20 (23.3)	0.98 (0.52, 1.87)
Nonbinary	22 (66.7)	1.72 (0.77, 3.84)	15 (45.5)	1.84 (0.84, 4.01)	10 (38.5)	2.63* (1.03, 6.68)
Transgender Men	7 (63.6)	1.55 (0.43, 5.58)	6 (54.6)	2.20 (0.63, 7.64)	4 (44.4)	3.21 (0.73, 14.0)
Transgender Women	11 (64.7)	1.55 (0.53, 4.55)	6 (35.3)	0.81 (0.28, 2.34)	1 (25.0)	0.60 (0.06, 6.20)
**Sexual Orientation**						
Asexual	7 (58.3)	1.34 (0.40, 4.52)	4 (36.4)	0.91 (0.25, 3.38)	1 (14.3)	0.69 (0.08, 6.26)
Bisexual, Pansexual, Queer	79 (59.0)	1.56 (0.94, 2.58)	42 (31.3)	0.83 (0.49, 1.39)	26 (27.7)	2.08 (0.97, 4.47)
Gay, Lesbian	95 (61.7)	1.48 (0.92, 2.39)	49 (31.6)	0.74 (0.45, 1.21)	33 (29.2)	1.57 (0.77, 3.20)
Straight (Heterosexual)	86 (53.8)	1 (reference)	65 (40.9)	1 (reference)	22 (24.2)	1 (reference)
Not sure	9 (75.0)	3.34 (0.84, 13.22)	6 (50.0)	2.19 (0.63, 7.59)	4 (66.7)	15.44** (2.22, 107.6)
**English Proficiency**						
English Proficient	201 (56.6)	1 (reference)	117 (33.0)	1 (reference)	66 (26.2)	1 (reference)
Limited English Proficiency	50 (57.5)	1.04 (0.61, 1.80)	40 (47.1)	1.66 (0.96, 2.88)	15 (39.5)	1.37 (0.62, 3.02)
**Health Literacy Level**						
High	219 (56.9)	1 (reference)	131 (34.1)	1 (reference)	68 (25.5)	1 (reference)
Low	65 (56.5)	1.00 (0.65, 1.57)	44 (38.6)	1.09 (0.69, 1.72)	21 (37.5)	1.96* (1.03, 3.74)

a. Row percentages were calculated out of total N based on number of respondents for the two variablesof interest.

b. aOR: Adjusted odds ratios were calculated by multivariable logistic regression adjusted for age andeducation.

c. Confidence Interval

d. The total n for job insecurity was 324, as 197 respondents did not answer the job insecurity question.These respondents were still included in the overall analysis as reports of other insecurities orhealthcare access issues remain important.

* p < 0.05

** p < 0.01

**Table 3:** Healthcare Access Outcomes by Demographics

Demographic Group	Uninsured (N, row %)	aOR (95% CI)	No primary care access (N, row %)	aOR (95% CI)	Has notseen aproviderin thelast year (N, row%)	aOR (95% CI)
Total N = 521	N = 120 (25.2)		N = 28 (7.0)		N = 188 (36.2)	
**Age Categories (Years)**						
18–24	26 (22.4)	1 (reference)	6 (6.1)	1 (reference)	54 (43.9)	1 (reference)
25–34	52 (22.4)	1.36 (0.78, 2.37)	11 (5.7)	1.24 (0.43, 3.56)	96 (37.9)	0.86 (0.54, 1.35)
35–44	24 (36.4)	2.42* (1.21, 4.82)	3 (5.8)	0.97 (0.22, 4.19)	15 (20.3)	0.31** (0.16, 0.62)
45+	17 (27.4)	1.03 (0.48, 2.18)	7 (13.2)	1.36 (0.39, 4.75)	23 (33.3)	0.55 (0.29, 1.05)
**Race**						
White	30 (20.6)	1 (reference)	9 (7.9)	1 (reference)	47 (31.3)	1 (reference)
Black or African American	7(31.8)	1.52 (0.55, 4.18)	1 (5.6)	0.50 (0.06, 4.45)	11 (44.0)	1.37 (0.57, 3.31)
Native American/Alaskan Native	15 (39.5)	3.01** (1.33, 6.84)	1 (2.9)	0.33 (0.04, 2.77)	12 (29.3)	0.85 (0.40, 1.83)
Asian	2 (66.7)	14.98* (1.13, 198.8)	0 (0)	n/a	2 (50.0)	2.2 (0.29, 16.3)
Native Hawaiian/Pacific Islander	0 (0)	n/a	0 (0)	n/a	0 (0)	n/a
Mixed	3 (11.1)	0.66 (1.78, 2.45)	0 (0)	n/a	9 (32.1)	1.18 (0.48, 2.88)
Other	38 (25.2)	1.06 (0.60, 1.87)	8 (6.7)	0.57 (0.20, 1.63)	66 (39.3)	1.35 (0.84, 2.18)
Did not respond	25 (28.4)	1.15 (0.60, 2.20)	9 (10.3)	0.94 (0.33, 2.69)	41 (40.2)	1.39 (0.80, 2.41)
**Origin**						
United States	37 (14.2)	1 (reference)	7 (2.9)	1 (reference)	103 (36.5)	1 (reference)
Puerto Rico	1 (25.0)	1.81 (0.18, 18.7)	0 (0)	n/a	0 (0)	n/a
Mexico	32 (27.8)	2.33** (1.30, 4.18)	7 (6.9)	1.54 (0.44, 5.45)	40 (30.5)	0.78 (0.48, 1.27)
Venezuela	30 (62.5)	6.82** (3.26, 14.28)	10 (33.3)	11.30** (3.19, 40.0)	24 (48.0)	1.82 (0.91, 3.65)
Other	13 (36.1)	3.87** (1.72, 8.71)	3 (13.6)	5.90* (1.29, 27.0)	16 (43.2)	1.55 (0.75, 3.21)
**Education**						
Less than high school	21 (43.8)	1 (reference)	10 (23.3)	1 (reference)	26 (44.8)	1 (reference)
Completed high school	78 (29.7)	0.52 (0.26, 1.02)	15 (7.0)	0.30* (0.11, 0.79)	107 (37.5)	0.62 (0.34, 1.14)
Bachelor's degree	16 (15.0)	0.20** (0.09, 0.46)	2 (2.2)	0.09** (0.02, 0.47)	37 (32.5)	0.49* (0.24, 0.98)
Advanced degree	5 (8.5)	0.11** (0.03, 0.33)	1 (1.9)	0.07* (0.01, 0.62)	17 (27.4)	0.38* (0.17, 0.85)
**Gender Identity**						
Cisgender Men	65 (27.8)	1 (reference)	17 (9.2)	1 (reference)	89 (35.7)	1 (reference)
Cisgender Women	27 (19.9)	0.64 (0.37, 1.10)	4 (3.3)	0.30* (.09, 0.96)	57 (38.3)	1.02 (0.65, 1.58)
Nonbinary	2 (6.5)	0.26 (0.06, 1.15)	1 (3.5)	0.56 (0.07, 4.66)	14 (38.9)	1.05 (0.50, 2.2)
Transgender Men	1 (11.1)	0.34 (0.04, 2.87)	0 (0)	n/a	2 (18.2)	0.33 (0.07, 1.62)
Transgender Women	5 (35.7)	0.87 (0.26, 2.90)	3 (20.0)	1.28 (0.29, 5.68)	5 (29.4)	0.61 (0.20, 1.90)
**Sexual Orientation**						
Asexual	4 (40.0)	2.10 (0.54, 8.21)	1 (11.1)	1.65 (0.17, 16.0)	5 (41.7)	1.34 (0.38, 4.63)
Bisexual, Pansexual, Queer	22 (17.2)	0.69 (0.37, 1.28)	2 (1.7)	0.31 (0.06, 1.51)	44 (31.9)	0.67 (0.40, 1.14)
Gay, Lesbian	32 (21.3)	0.84 (0.48, 1.47)	4 (3.2)	0.47 (0.14, 1.60)	49 (31.2)	0.76 (0.46, 1.25)
Straight (Heterosexual)	44 (29.7)	1 (reference)	10 (8.6)	1 (reference)	60 (36.8)	1 (reference)
Not sure	4 (30.8)	1.40 (0.39, 5.05)	2 (28.6)	4.83 (0.70, 33.5)	4 (30.8)	0.79 (0.22, 2.77)
**English Proficiency**						
English Proficient	65 (18.9)	1 (reference)	15 (5.1)	1 (reference)	124 (33.9)	1 (reference)
Limited English Proficiency	45 (57.0)	4.33** (2.42, 7.77)	12 (22.2)	2.90* (1.10, 7.69)	40 (44.0)	1.66 (0.96, 2.87)
**Health Literacy Level**						
High	82 (21.9)	1 (reference)	17 (5.3)	1 (reference)	128 (32.1)	1 (reference)
Low	38 (37.6)	1.80* (1.10 2.95)	10 (13.3)	1.89 (0.77, 4.67)	58 (49.2)	1.90** (1.22, 2.97)
